# Characterization of size distribution and markers for mosquito extracellular vesicles

**DOI:** 10.3389/fcell.2025.1497795

**Published:** 2025-04-11

**Authors:** Félix Rey-Cadilhac, Florian Rachenne, Antonin Marquant, Josephine Lai Kee Him, Aurélie Ancelin, Veronica Foisor, Marie Morille, Sébastien Lyonnais, Chantal Cazevieille, Dorothée Missé, Julien Pompon

**Affiliations:** ^1^ MIVEGEC, Univ. Montpellier, IRD, CNRS, Montpellier, France; ^2^ ICGM, Univ. Montpellier, CNRS, ENSCM, Montpellier, France; ^3^ CBS (Centre de Biologie Structurale), Univ. Montpellier, CNRS, Inserm, Montpellier, France; ^4^ Unchained Labs, Royston, United Kingdom; ^5^ Institut Universitaire de France (IUF), Paris, France; ^6^ CEMIPAI, UAR3725 CNRS - Montpellier University, Montpellier, France; ^7^ INM (Institut de Neuroscience de Montpellier), Electronic Microscopy Plateform, Saint Eloi Hospital, Montpellier, France

**Keywords:** extracellular vesicles, mosquito, microscopy, protein markers, syntenin, tetraspanin, characterization, analytical methods

## Abstract

Extracellular vesicles (EVs) are non-replicative, cell-derived membranous structures secreted by potentially all eukaryotic cells, playing a crucial role in intercellular communication. The study of EVs requires approaches and tools, which have predominantly been developed for mammalian models. Here, we undertook a multimodal characterization of mosquito EVs to provide a technical and knowledge foundation for their study. First, using a cell line model from *Aedes aegypti* and applying multiple analytical technologies (i.e., NTA, TEM, cryo-EM, and AFM), we observed that mosquito EVs range from 20 to 500 nm in diameter and that a majority are smaller than 100 nm. Second, we showed that smaller EVs are secreted in mosquito saliva. Third, we evaluated the capacity of differential centrifugation and size exclusion chromatography to separate mosquito EVs, revealing the strengths and weaknesses of each technology. Finally, we identified a mosquito homolog of CD63 as an extravesicular marker and the mosquito syntenin as a putative luminal marker. Overall, our results promote the development of tools and approaches for the study of mosquito EVs.

## 1 Introduction

Extracellular vesicles (EVs) are “particles naturally released from the cell that are delimited by a lipid bilayer” (Théry et al., 2018). Observed for the first time during the late 70s (Wolf, 1967), EVs were initially considered a cell waste disposal system until multiple studies revealed their capacity to act as cell–cell vehicles. As a result, EVs impact diverse biological processes through the delivery of proteins, lipids, and/or nucleic acids to recipient cells (Couch et al., 2021). Recent technological improvements unraveled a wide diversity of EV types, with variable size, density, cargo content, and membrane lipid and protein compositions (van Niel et al., 2018; Raposo and Stoorvogel, 2013).

Three types of mammalian EVs, namely, apoptotic bodies, ectosomes [i.e., microvesicles (MVs) and oncosomes], and exosomes, have been documented. These different types are distinguished based on their size, biochemical characteristics, and, most importantly, biogenesis mechanism (van Niel et al., 2018). Apoptotic bodies are the largest EVs, ranging from 500 nm to several micrometers (Battistelli and Falcieri, 2020). They originate from apoptosis and are generally rapidly phagocytosed by surrounding cells (Battistelli and Falcieri, 2020). The biological function of apoptotic bodies remains largely uncharacterized. Ectosomes range from less than 100 nm to several micrometers and originate from the budding of the cellular plasma membrane (Colombo et al., 2013). Ras homologous GTPases (RhoGTPases), particularly RhoA, are involved in ectosome secretion (Colombo et al., 2013). Finally, exosomes are the smallest EVs and range from 50 to 150 nm (Mateescu et al., 2017). Their biogenesis starts with invagination of late endosome membranes to form multivesicular bodies (MVBs), encompassing intraluminal vesicles (ILVs). MVBs then fuse with the plasma membrane to release ILVs as exosomes in the extracellular space (Kestens et al., 2017; Jeppesen et al., 2019). The formation of MVBs is orchestrated by proteins from the endosomal sorting complex required for transport (ESCRT) (Colombo et al., 2013) but requires other proteins such as syntenin. By interacting with syndecans, via PDZ domains, syntenin recruits ALG-2-interacting protein-X (ALIX) through LPXY(n)L motifs (Vora et al., 2018; Yeh et al., 2023; Reyes-Ruiz et al., 2019). In addition to its role in exosome biogenesis, syntenin participates in maintaining the EV structure by interacting with tetraspanins, which maintain the EV membrane and are required for EV secretion (Vora et al., 2018; Hitakarun et al., 2022).

The different technologies to separate/concentrate EVs leverage EV biophysical specificities (Théry et al., 2018). The most commonly used strategy applies differential centrifugation to separate EV populations according to their sedimentation rates. Density-based separation can also be achieved using the density gradient. Separation by size can be obtained by concentrating EVs using ultrafiltration before applying size-exclusion chromatography (SEC) (Mateescu et al., 2017). During SEC, the EV solution is eluted through a gel composed of beads harboring micropores. As the sample flow through, smaller EVs enter the pores, delaying their elution, whereas larger EVs that cannot enter the pores are eluted more quickly within the earlier fractions.

Several light-scattering/microscopic devices are used to observe EVs (Théry et al., 2018). Nanoparticle tracking analysis (NTA) allows single-particle measurement analysis using video recording of light scattered by nanoparticles undergoing Brownian motion. In contrast to dynamic light scattering (DLS) which studies Brownian motion (diffusion coefficient) of bulk particles, NTA analyzes individual particles, allowing the identification of their hydrodynamic diameter and their concentrations. It is consequently assumed to be less prone to interference caused by aggregates or larger particles than the DLS technique. Moreover, this method was validated for the size determination of synthetic nanoparticles following quality criteria if particles are superior to 50 nm (Kestens et al., 2017). Transmission electron microscopy (TEM) and cryo-electron microscopy (cryo-EM) are used to visualize EV sizes and can reveal lipid bilayers, which structurally define EVs. Atomic force microscopy (AFM) enables topographic analysis to measure the width and height of EVs in three dimensions. Moreover, EV-enriched proteins are used as markers for immunoprecipitation and immunostaining (Théry et al., 2018). Most preponderant mammalian markers include transmembrane tetraspanins (e.g., CD63 and CD81) and cytosolic proteins recovered in EVs (e.g., syntenin and Alix) (Théry et al., 2018). However, association between specific markers and EV types remains to be solidly supported (Théry et al., 2018; Kestens et al., 2017).

Although our knowledge about EVs, their compositions, and functions has exponentially increased during the last two decades, most of the data concern mammalian EVs. EVs are supposedly secreted by most cell types (van Niel et al., 2018) and were predictably visualized in mosquito cell media (Vora et al., 2018) and mosquito saliva (Yeh et al., 2023). A combination of studies using TEM, cryo-EM, and AFM revealed that cells derived from *Aedes albopictus* mosquito harbor structures similar to MVBs and secrete EVs ranging from 50 to 250 nm (Vora et al., 2018; Reyes-Ruiz et al., 2019). Providing further support to EV secretion by mosquitoes, we reported the presence of lipid bilayer vesicles between 85 and 900 nm in saliva from *Aedes aegypti* mosquitoes (Yeh et al., 2023). Homologs of human tetraspanins such as CD63, CD9, CD81, or Alix have been proposed as markers for mosquito EVs (Vora et al., 2018; Hitakarun et al., 2022). However, these prior studies used antibodies against human homologs and did not validate the mosquito targets. There is, therefore, a lack of knowledge about mosquito EV size distribution and an absence of validated markers that hinders mechanistic characterization of EV function in mosquitoes, particularly EV function in viral disease transmission by mosquitoes (Rey-Cadilhac et al., 2023; Sultana and Neelakanta, 2020; Chávez et al., 2019).

EVs from other arthropod models were described in the literature. Recently, EVs from the *Spodoptera frugiperda* insect’s cell line Sf9 were observed with TEM after SEC separation, and their size was estimated between 80 and 100 nm using NTA (Van Es et al., 2024). No EV marker detection was performed during this study (Van Es et al., 2024). For the tick model, EVs have been detected in the ISE6 cell line through cryo-EM with a predominant population of EVs exhibiting diameters between 50 and 100 nm (Zhou et al., 2018). Subsequent studies have identified the presence of EV markers in *Rhipicephalus haemaphysaloides* and *Hyalomma asiaticum* ticks. NTA revealed that EVs isolated from tick hemolymph have an average size of 100 nm (Xu et al., 2023). Proteomic profiling of these vesicles identified several key markers, including heat shock proteins, annexins, and proteasome subunits (Xu et al., 2023). Additionally, Western blot analysis using antihuman homologous antibodies confirmed the presence of TSG101 and CD9 as EV markers (Xu et al., 2023). Despite these advances, the comprehensive characterization of insect-derived EVs in general—including size distribution and validated marker profiles—remains an underexplored area.

Here, we characterized EVs secreted by cells and in saliva from *Aedes aegypti*, the main mosquito vector of multiple viral diseases (Pierson and Diamond, 2020). We undertook a thorough description of EV size distribution using multiple microscopic technologies and evaluated the capacity of SEC to separate EV populations. Finally, we identified mosquito EV markers. In our endeavor, we abided by the rules set forth by the EV community in their paper on the Minimal Information for Studies of Extracellular Vesicles (MISEV), published in 2014 (Lötvall et al., 2014), updated in 2018 (Théry et al., 2018), and still undergoing refinements (Witwer et al., 2021; Welsh et al., 2024). These rules include the general denomination of secreted vesicles as EVs until a specific type is ascribed based on the characterized biogenesis, the use of multiple microscopic technologies to evaluate size, and the dual identification of extravesicular and lumen markers. By expanding our knowledge about mosquito EVs, our results will foster the study of EV biology and function in entomological models.

## 2 Material and methods

### 2.1 Cells

The *Aedes aegypti* Aag2 cell line, derived from whole homogenized embryos (Lan and Fallon, 1990), was grown in Roswell Park Memorial Institute (RPMI) medium (Gibco) with 10% decomplemented fetal bovine serum (FBS) (Gibco), 1× nonessential amino acids (Gibco), and 1% penicillin/streptomycin (Gibco) at 28°C with 5% CO_2_. Prior EV collection, complete medium was replaced by RPMI medium with 2% EV-depleted FBS, 1× nonessential amino acids, and 1% penicillin/streptomycin. EV depletion from decomplemented FBS was obtained by ultracentrifugation at 100,000 g for 18 h using an S58-A rotor (k-factor 50, Thermo Scientific) in Sorvall MX Plus Series Floor Model Micro-Ultracentrifuge (Thermo Scientific). The EV-depleted supernatant was collected by leaving approximately 1 mL at the bottom of the 8-mL tube, filtered through a 0.22-µm filter (Sartorius), and stored at 4°C.

### 2.2 Mosquitoes

*Aedes aegypti* mosquitoes from the “Bora bora” colony collected in French Polynesia in 1980 (Kuno, 2010) were reared in cages (BugDorm) at 27°C ± 1°C, 70% ± 5% relative humidity, with a 12 h:12 h day:night cycle, and *ad libitum* access to 10% sugar solution. After egg hatching, larvae were reared at 28°C in MilliQ water supplemented with yeast and fish food flakes (TetraMin) until pupation.

### 2.3 EV separation from cell culture

Aag2 cells (5 × 10^6^) in T75 flasks (Thermo Scientific) were reared in 8 mL of EV-depleted FBS media, or 1.8 × 10^7^ Aag2 cells were reared in 16 mL of EV-depleted FBS media. As control, unconditioned media (UCM) was subjected to the same procedure without cells. Following 24, 48, or 72 h after changing the media, cell medium was collected and precleared by centrifugation at 1,500 (for NTA, SEC, AFM, and Leprechaun analyses) or 3,500 g (for TEM and cryo-EM analyses) for 10 min at 4°C.

To perform differential centrifugation, the 8-mL precleared cell medium was centrifuged at 10,000 g for 30 min at 4°C, and the pellet was resuspended in 500 µL of PBS. The resulting supernatant was ultracentrifuged at 100,000 g for 3 h at 4°C, and the pellet was resuspended in 7 mL of PBS, followed by performing ultracentrifugation a second time at 100,000 g for 3 h at 4°C. The final pellet was resuspended in 250 µL of PBS for NTA and AFM observations.

EV precipitation by ultracentrifugation of the precleared cell medium was conducted at 100,000 g for 3 h at 4°C using an S50-ST swing-bucket rotor (k-factor 76.6, Thermo Scientific) in Sorvall MX Plus Series Floor Model Micro-Ultracentrifuge. The pellet was washed with 7 mL of PBS (Gibco) and ultracentrifuged again at 100,000 g for 3 h at 4°C. The final pellet was resuspended in 30 µL of PBS for cryo-EM and in 500 µL of PBS for TEM observations.

### 2.4 EV separation from mosquito saliva

Ten-day-old female mosquitoes were starved for 4 h and offered to feed on 3 mL of PBS covered with a silicone membrane for 2 h using a membrane feeding system (Hemotek). The solution was then precleared by centrifugation at 1,500 g for 10 min at 4°C and subjected to ultracentrifugation at 100,000 g for 3 h at 4°C. The pellet was resuspended with 30 µL of PBS for TEM analyses.

### 2.5 Nanoparticle tracking assay

EV samples from Aag2 cells were diluted to approximately 2 × 10^8^ particles per ml in particle-free PBS and were analyzed using NanoSight NS300 (Malvern) with a 488-nm laser wavelength using the following capture settings: camera type: sCMOS, laser type: Blue488, camera level: 15, slider shutter: 1206, slider gain: 366, FPS: 25.0, number of frames: 1498, temperature: 25.0°C–25.0°C, viscosity (water 0.9 cP), and syringe pump speed: 40. Five 1-min films were taken, and the analysis was performed using NanoSight NTA 3.4 Build 3.4.4 software (Malvern) with a detection threshold set to 5. Only particle sizes found in at least two films were considered. Particles detected in negative UCM control were subtracted from the results for concentration measurements. Analyses were performed at the Institute Charles Gerhardt Montpellier, Montpellier, France.

### 2.6 Transmission electronic microscopy

Seven microliter of the ultracentrifuged EV solution was loaded onto a formvar-coated copper grid 100 mesh (EMS FCF100-Cu-50), left to settle for 2 min, and dried with a blotting paper. The grid was stained with 1% uranyl acetate and analyzed using a TEM F20 operating at 120 KV (Tecnai) with a Veleta numeric camera (Olympus) at the Neuroscience institute of Montpellier, INSERM U1298, France.

### 2.7 Cryo-electron microscopy

Three microliter of the EV solution was loaded onto a polarized Lacey Carbon-Supported Copper Grid (Electron Microscopy Sciences) in a Leica EMGP2 chamber (Leica Microsystems) at 10°C with a 95% humidity rate, dried for 3 s on a blotting paper, frozen in ethanol, and stored in liquid nitrogen. The grid was observed using the TEM JEOL 2200FS (JEOL, EUROPE) with a K3 camera (Gatan-Ametek, United States) and analyzed using DigitalMicrograph software (GMS 3).

### 2.8 Atomic force microscopy

Freshly cleaved muscovite mica sheets (V1 grade, Ted Pella Inc.) were glued on a glass slide, coated overnight at 4°C with 0.01% poly-L-lysine (Sigma), rinsed with 3 mL of MilliQ water, and dried using an N2 flux. A 3D-printed O-ring was then glued on the glass slide to assemble a small liquid cell. The separated EV solution was diluted 10-fold in buffer A (20 mM Tris HCl pH 7.5 and 100 mM NaCl), and 200 μL was deposited on the liquid cell to allow passive particle adsorption. The cell was rinsed with 200 µL of buffer A, and 200 μL of buffer A were added before imaging. Topographic imaging was performed in the quantitative imaging (QI) mode, which is a force-curve-based imaging mode, using Sharp Nitride Lever (SNL) B probes (mean cantilever spring constant kcant = 0.11 N/m, Bruker). The applied force was kept at 450 pN and a constant approach/retract speed of 75 μm/s (z range of 300 nm). Imaging was performed at room temperature using an AFM NanoWizard 4xp instrument (JPK BioAFM, Bruker Nano GmbH, Berlin, Germany) operating in the BSL3 of CEMIPAI facility (UAR3725 CNRS and Montpellier University, France). Picture analyses were performed using JPK Data Processing software (7.0.165 version).

### 2.9 Size-exclusion chromatography

The precleared supernatant was concentrated from 8 to 2 mL by centrifugation at 3,739 g for 5 min at 4°C with a 10-kDa filter (Amicon Ultra-15, PLGC, membrane Ultracel-PL, Merck) and centrifuged at 10,000 g for 10 min at 4°C to remove large particles. An aliquot of the resulting supernatant was analyzed using NTA. The remaining solution was completed to 2 mL with degassed PBS and separated using SEC with a qEV2/35-nm GEN 2 column (IZON) and automatic fraction collector v2 (IZON) using the following parameters: 10 fractions of 1 mL with a default buffer volume. Each fraction was analyzed using NTA after overnight storage at 4°C and concentrated from 500 to ∼50 µL by centrifugation at 10,000 g for 1 h at 4°C with a 3-kDa filter (Amicon Ultra-0,5, Membrane Ultracel-3, PMNL, Merck) before Western blot (WB) analysis.

### 2.10 Amino acid phylogenetic analysis

Sequences from human and *Aedes aegypti* tetraspanins were obtained from UniprotKB and VectorBase, respectively. Alignment and tree were performed with MEGA11 using the maximum likelihood statistical method.

### 2.11 DsRNA-mediated RNAi

Target sequences were amplified from Aag2 cDNA using GoTaq Master mix (Promega) and 400 nM of T7 sequence-flanked primer pairs (Supplementary Table S1) designed using the E-RNAi website (Horn and Boutros, 2010). The PCR thermal profile was 95°C for 5 min; 45 cycles of 95°C for 15 s, 55°C for 15 s, and 72°C for 45 s; and a final elongation at 72°C for 10 min. After verifying the size on agarose gel, PCR products were purified using the PCR QIAquick kit (Qiagen) and transcribed overnight using the MegaScript T7 kit (Thermo Fisher). RNA was extracted using the EZNA total RNA kit (Omega), adjusted to 1 μg/μL, and folded by heating at 95°C for 5 min followed by slow cooling. Negative control dsRNA targeting LacZ was similarly produced. A total of 250,000 Aag2 cells plated in P24-well plates were transfected with 0.5 µg of dsRNA using the TransIT-mRNA Transfection Kit (Mirus) for 24 h. After transfection, the media was replaced with 250 µL of RPMI with 2% EV-depleted FBS, 1% penicillin/streptomycin, and 1× nonessential amino acids.

### 2.12 Gene expression quantification

Seventy-two hours post dsRNA transfection, cells were lysed with 350 µL of TRK lysis buffer and RNA was extracted using the EZNA total RNA extraction kit. RNA was DNase-treated, normalized after nanoDrop quantification (ThermoFisher Scientific), and reverse transcribed using the iScript gDNA Clear cDNA Synthesis Kit (Biorad). Gene expression was quantified using Hot firepol SolisGreen qPCR mix (Euromedex) with 300 nM of forward and reverse primers (Supplementary Table S1) in a total volume of 10 µL. The thermal profile was 95°C for 5 min and 40 cycles of 95°C for 15 s, 60°C for 20 s, and 72°C for 20 s, with a final melting curve analysis in LightCycler 96 (Roche). *Actin* expression was used as a housekeeping gene. Relative expression was quantified using the delta–delta Ct method.

### 2.13 Western blot

Cell media was centrifuged at 3,000 g for 5 min and kept at −20°C. Cells were lysed with RIPA buffer, incubated on ice for 20 min, and centrifuged at 20,000 g for 20 min before the supernatant was collected. After protein quantification, normalized protein amounts of cell media and lysed cells were loaded on the gel. For EVs concentrated by differential centrifugation and SEC, volumes of pellets and supernatants and volumes of fractions and input were normalized to enable comparison. Cell media, concentrated EV solution, and cell lysate were diluted in 1X reducing Laemmli SDS sample buffer (ThermoScientific), heated at 95°C for 10 min, and loaded on NuPAGE 12%, Bis–Tris gel (Invitrogen) for migration at 120 V for 5 min and then 150 V for 1 h. Protein was transferred onto a nitrocellulose membrane (Bio-Rad) using a Trans-Blot Turbo RTA Mini 0.2 µm Nitrocellulose Transfer Kit (Bio-Rad) and a Trans-Blot Turbo Transfer System (Bio-Rad). Membranes were stained with red ponceau (Sigma) for 5 min. After rinsing in 1× PBS 0.1% Tween, membranes were blocked with 1× PBS 0.1% Tween 20 5% milk (Régilait) for 45 min and incubated with 1:1000 antihuman CD63 (Ab134045, clone EPR5702, Abcam), 1:400 antihuman pan-actin (MA5-11869, clone ACTN05, ThermoScientific), or custom-made anti-*Aedes aegypti* syntenin antibody at 1:500 for cell media, at 1:200 for separated EV solutions, and at 1:2000 for cell lysate in 1× PBS 0.1% Tween 20, overnight at 4°C. Anti-*Aedes aegypti* syntenin antibody was generated by Proteogenix (Strasbourg, France) using the CTSFIRGKMDHSVPD peptide sequence (aa 316–330) in rabbit. Secondary antibody staining was performed with 1:2000 HRP-conjugated anti-rabbit IgG (H + L) (7074, Cell Signaling Technology) and 1:2000 HRP-conjugated anti-mouse IgG (H + L) (7076, Cell Signaling Technology) diluted in 1× PBS 0.1% Tween 20 5% milk and incubated for 1 h at room temperature. Blots were developed using SuperSignal West Pico PLUS Chemiluminescent Substrate (ThermoScientific) with the ChemiDoc MP Imagin System (Bio-Rad). Images were analyzed using ImageLab Software (Bio-Rad).

### 2.14 Leprechaun analysis

Samples were analyzed using the Leprechaun Exosome Human Tetraspanin Flex Kit (Unchained Labs, US) conjugated with hCD63 (Ab134045, clone EPR5702, Abcam) and MIgG control (400101, clone MOPC-21, Biolegend), following manufacturer’s instructions. Precleared cell media was diluted in the manufacturer-supplied incubation solution and incubated for 1 h at RT on the functionalized Tetraspanin Flex Lunis. Lunis were then processed on the Luni Washer (Unchained Labs, US) using the exosome protocol, dried, and imaged using the Leprechaun instrument with Leprechaun Client v2.0. Data were analyzed using Leprechaun Analysis v 2.0 with the automatic fluorescent cutoffs applied.

### 2.15 Statistics

Gene expression changes were assessed with *post hoc* multiple comparison Fisher’s LSD tests. Statistical analyses were performed using Prism 9.0 (GraphPad).

## 3 Results

### 3.1 Detection and size distribution of EVs secreted by mosquito cells

**FIGURE 1:**
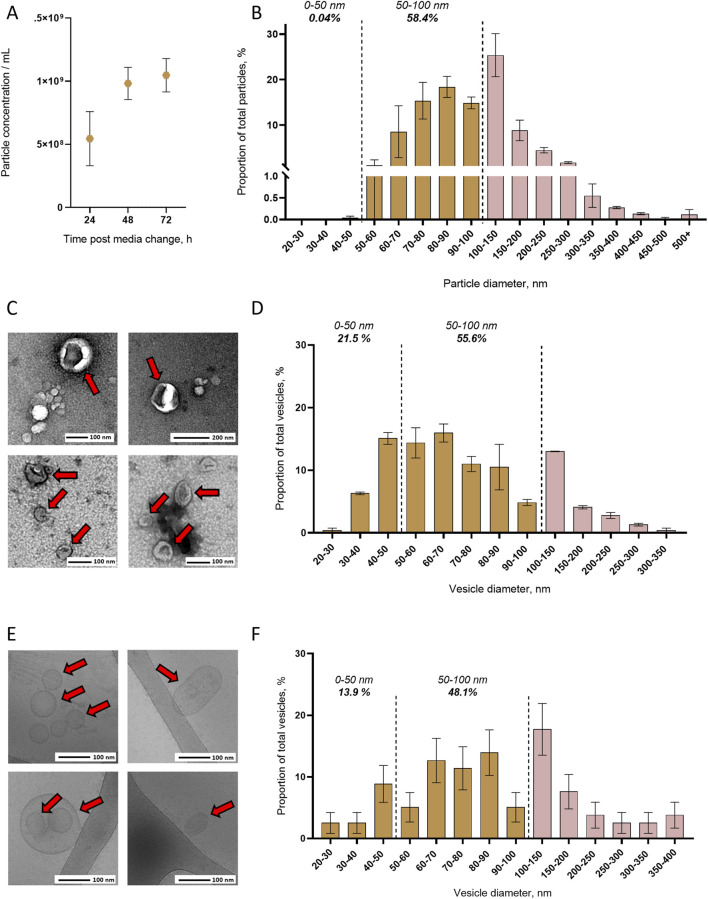
Detection and size distribution of extracellular vesicles released from mosquito cells. **(A)** Quantification of particles in cell media with NTA at 24, 48, and 72 h after changing cell media. N, two biological repeats. **(B)** Size distribution of total particles in cell media by NTA at 72 h after changing cell media. Total N EVs, 1.05 × 10^9^ from two biological repeats. **(C, D)** Representative pictures **(C)** and size distribution **(D)** with TEM of EVs concentrated by ultracentrifugation. N analyzed EVs, 223 from two biological repeats. **(E, F)** Representative pictures **(E)** and size distribution **(F)** with cryo-EM of EVs concentrated by ultracentrifugation. N analyzed EVs, 79 from two biological repeats. **(A, B, D, and F)** Lines and bars indicate mean ± s.e.m.

To initiate the quantification of EVs secreted from mosquito cells, we evaluated the dynamic of particle secretion using NTA on cell media collected every 24 h at 24, 48, and 72 h post media replacement. We intentionally used the term particles to describe objects detected with NTA as the technology could also quantify non-vesicular objects. Accordingly, in the unconditioned media (UCM), we detected particles ranging from 20 to 380 nm in diameter (Supplementary Figure S1) and subtracted this background from the corresponding size-matched particles in the cell media. Throughout our kinetic, we quantified an increasing number of particles starting from 5.45 × 10^8^ particles/mL at 24 h, 9.83 × 10^8^ particles/mL at 48 h, and up to 1.05 × 10^9^ particles/mL at 72 h (Figure 1A). We then described the particle size distribution at the peak of secretion at 72 h (Figure 1B; Supplementary Figures S2A, B). Particles ranged from 50 to more than 500 nm in diameter, with close to none below 50 nm, 58.4% between 50 and 100 nm, and 25.4% between 100 and 150 nm. We found an average size of 112 ± 7 nm and a mode of 80–90 nm.

To determine whether secreted particles are EVs and further describe their size distribution, we applied TEM and cryo-EM to EVs ultracentrifuged from cell media collected at 72 h. As for NTA, we first analyzed UCM with TEM and did not observe EVs but only protein aggregates (Supplementary Figure S3), providing a rationale for the detection of non-vesicular objects with NTA (Supplementary Figure S1). In cell media, TEM revealed enclosed cup-shaped structures typical of EVs (Figure 1C; Supplementary Figure S4A). EV size distribution as observed with TEM ranged from 20 to 350 nm in diameter, with 21.5% below 50 nm, 55.6% between 50 and 100 nm, and 13% between 100 and 150 nm. The average EV size was 82 ± 3 nm, and the mode was 60–70 nm (Figure 1D). Using cryo-EM, we observed vesicles delimited by a lipid bilayer (Figure 1E), which is a characteristic feature of EVs. Of note, we found EVs varying in shapes from round to more complex ovoid forms (Supplementary Figure S4B), and we could not determine whether this is a particularity of mosquito EVs or an artefact inherent to our experimental design. Size distribution of EVs observed with cryo-EM varied from 20 to 400 nm in diameter, with 13.9% below 50 nm, 48.1% between 50 and 100 nm, and 17.7% between 100 and 150 nm (Figure 1F). The average EV size was 115 ± 9 nm, and the mode was 80–90 nm. Of note, in both TEM and cryo-EM images, we observed non-vesicular aggregates and a few 70–80 nm hexagonal particles, which resemble virions (Figures 1C, E; Supplementary Figures S4C, D). Overall, we established that mosquito cells secrete EVs of variable sizes, mostly ranging from 50 to 100 nm, as determined by NTA and two microscopic technologies.

### 3.2 Size distribution of EVs in *Aedes aegypti* mosquito saliva

**FIGURE 2:**
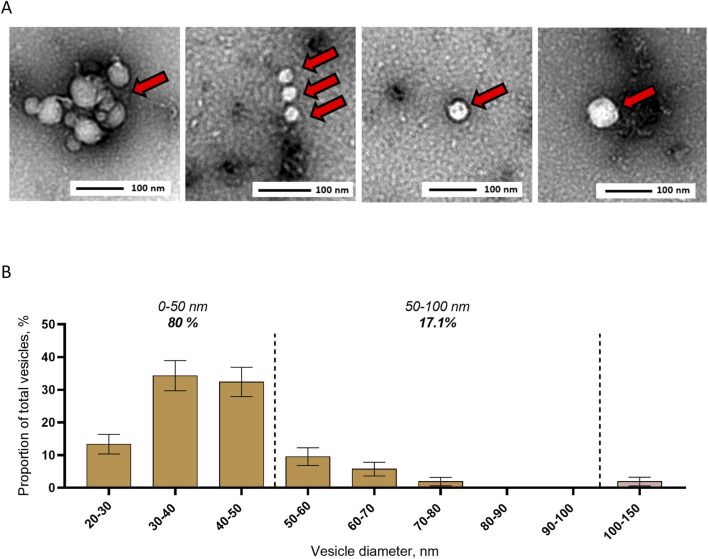
Detection and size distribution of extracellular vesicles secreted in saliva from *Aedes aegypti* female mosquitoes. **(A, B)** Representative pictures **(A)** and size distribution **(B)** with TEM of EVs separated by ultracentrifugation from pools of mosquito saliva. Bars indicate mean ± s.e.m. N analyzed EVs, 105 from two biological repeats.

To describe EVs in mosquito saliva, we collected saliva pools, concentrated EVs by ultracentrifugation, and applied TEM. Images showed EVs (Figure 2A) ranging from 20 to 150 nm in diameter, with 80% below 50 nm, 17.1% between 50 and 100 nm, and 2.9% between 100 and 150 nm for an average size of 43 ± 1 nm and a mode of 30–40 nm (Figure 2B). Our TEM analysis confirmed the secretion of EVs in mosquito saliva and indicated a relatively small size of salivary EVs.

### 3.3 Size separation of EVs using differential centrifugation and SEC

**FIGURE 3:**
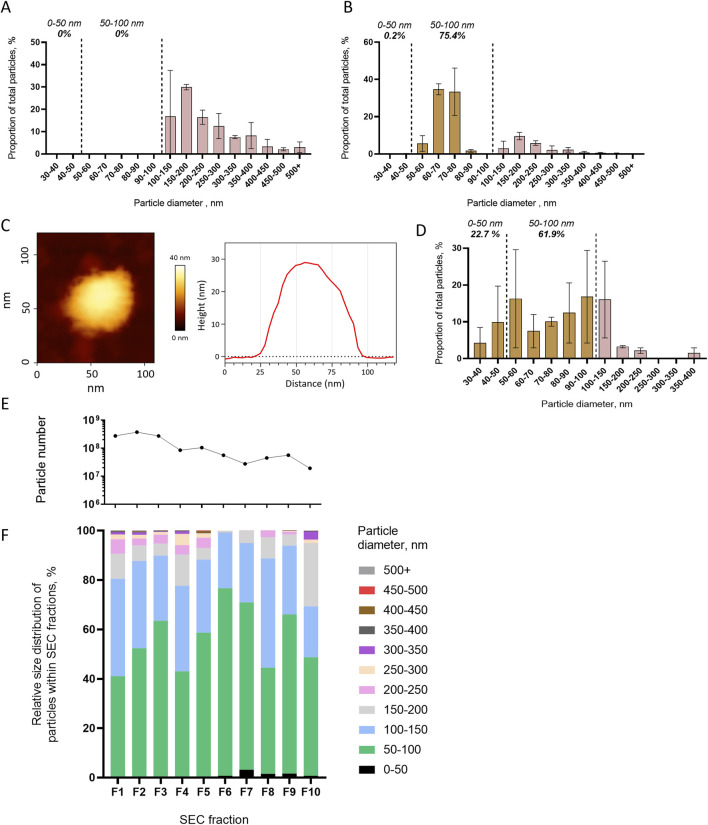
Differential centrifugation and SEC separation of extracellular vesicles released from mosquito cells. **(A, B)** Size distribution with NTA of particles pelleted at 10,000 g **(A)** and at 100,000 g **(B)**. **(C, D)** Representative picture and topography analysis **(C)** and size distribution **(D)** with AFM of particles pelleted at 100,000 g. N analyzed EVs, 176 from two biological repeats. **(E, F)** Particle number per fractions of SEC **(E)** and relative size distribution of particles in each fraction (F1–F10) **(F)** measured using NTA. One repeat is presented here, and a second one is presented in Supplementary Figure S6. **(A, B, D)** Bars indicate mean ± s.e.m.

We first tested whether centrifugation at 10,000 g and 100,000 g pelleted different size populations of mosquito cell EVs. EVs from mosquito cell media were separated by differential centrifugation, and the resulting pellets were analyzed by NTA. Centrifugation at 10,000 g isolated larger EVs (mean size of 243 ± 25 nm and mode of 150–200 nm) with no particles detected below 100 nm (Figure 3A). Subsequent 100,000 g ultracentrifugation concentrated smaller EVs, with 75.4% of the particles ranging from 50 to 100 nm, with a mean size of 106 ± 32 nm and a mode of 60–70 nm (Figure 3B). We further characterized the size distribution of the EVs pelleted at 100,000 g by using AFM. Topographic cartography of EVs enabled the measurement of width and height of each vesicle (Figure 3C; Supplementary Figures S5A–C), which were combined to calculate an estimated 3D diameter (Kanno et al., 2002). Size distribution of the pelleted EVs as measured with AFM showed that most of the particles were smaller than 150 nm, with 22.7% ranging from 30 to 50 nm, 61.9% ranging from 50 to 100 nm, and 16% ranging from 100 to 150 nm, for an overall mean size of 75 ± 3 nm (Figure 3D).

Second, we assessed the capacity of SEC to separate EVs by size. Ultrafiltrated EVs from mosquito cell media were fractionated in 10 SEC fractions, in which we analyzed the EV size distribution by NTA. Although higher quantities of particles were detected in the first fractions, the size distribution was not altered across the fractions (Figures 3E, F). The most abundant 50- to 100-nm size range of EVs (Figure 1B) represented the majority in all fractions, and there was neither depletion in a smaller size range nor enrichment in larger EVs with the increasing number of fractions (Figure 3F). Although slightly variable, the trends were similar in a biological repeat (Supplementary Figures S6A, B).

### 3.4 Identification of lumen and transmembrane EV markers

We evaluated syntenin and the mosquito homologs of human CD63 (hCD63) as EV markers. Although *A. aegypti* syntenin (AAEL0005391) is functionally uncharacterized, comparison with the human protein sequence showed a moderate homology with 46% identities and 65% similarities, and a good conservation of the functional PDZ domains and LYPX(n)L motifs (Table 1). Furthermore, we found putative homologs of the syntenin partners such as syndecan and ALIX in the *A. aegypti* genome (Supplementary Table S2). To identify hCD63 homologs in *A. Aegypti*, we built a maximum likelihood amino acid tree (Supplementary Figure S7) and selected Tsp29Fa (AAEL003210) and Tsp29Fb (AAEL005536) as putative homologs. Tsp29Fb was previously identified as the hCD63 homolog (Vora et al., 2018); however, the AAEL annotation has changed ever since.

**TABLE 1:** Conservation of protein domains between human and *Aedes aegypti* syntenin.

			Human	*A. aegypti*
Protein		ID	Syntenin 1	AAEL005391
		Length, aa	298	331
		Identity, %	46	
		Similarities, %	65	
		Gaps, aa	33	
Domain	PDZ-1	Identity, %	57	
		Similarities, %	74	
		Gaps, aa	0	
	PDZ-2	Identity, %	64	
		Similarities, %	83	
		Gaps, aa	0	
	LYPX(n)L motif 1		3_LYPSL_7	3_LYPSL_7
	LYPX(n)L motif 2		45_LYPSL_49	76_FYPDL_80

**FIGURE 4:**
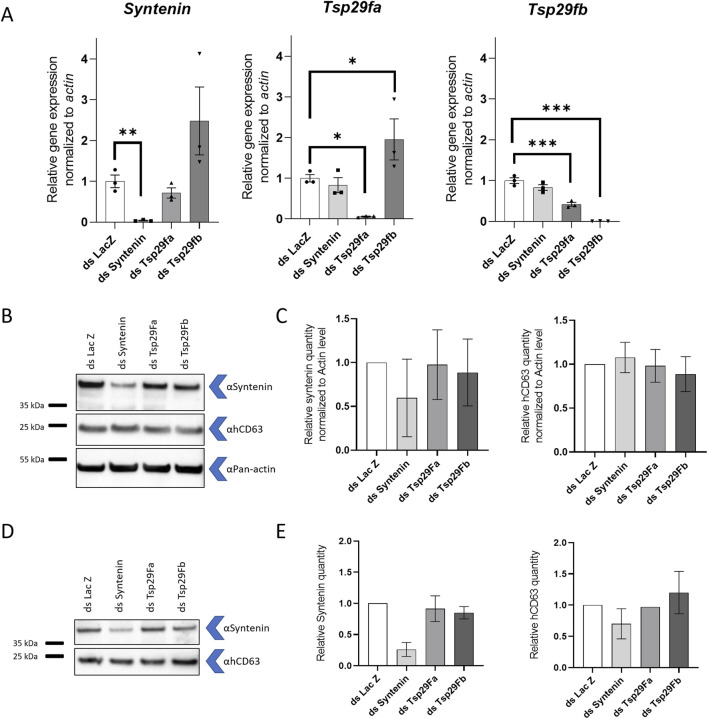
Detection of mosquito syntenin and human CD63 homolog. **(A)** Gene expression of *syntenin*, *Tsp29fa*, and *Tsp29fb* at 72 h post transfection with dsRNA against *syntenin* (dsSyntenin), *Tsp29Fa* (dsTsp29fa), and *Tsp29Fb* (dsTsp29fb). DsRNA against LacZ was used as control. **(B, C)** Representative WB **(B)** and quantification **(C)** of cellular mosquito syntenin and hCD63 homologs at 72 h post transfection with dsRNA against *syntenin*, *Tsp29fa*, and *Tsp29fb*. Pan-actin was used as loading control. N, 2. **(D, E)** Representative WB **(D)** and quantification **(E)** of secreted mosquito syntenin and hCD63 homologs at 72 h post transfection with dsRNA against *syntenin*, *Tsp29fa*, and *Tsp29fb*. **(A, C, E)** Bars indicate mean ± s.e.m. **(A)** Each point indicate a repeat. *, p < 0.05; **, p < 0.01; ***, and p < 0.001, as determined by Fisher LSD’s test. **(C, E)** N, 2.

To validate antibodies for these two mosquito EV markers, we separately depleted mosquito *syntenin*, *Tsp29Fa*, and *Tsp29Fb* through RNAi in mosquito cells and quantified the effect on protein levels using WB. To detect syntenin, we used a custom-made antibody targeting *A. aegypti* syntenin, whereas we used a human-targeted commercial CD63 antibody to tentatively detect Tsp29Fa and/or Tsp29Fb. DsRNA-mediated depletion significantly reduced the expression of the targeted genes (Figure 4A) and did not alter cell survival (Supplementary Figure S8). Of note, dsRNA against *Tsp29Fb* also decreased mRNA levels for *Tsp29Fa* (Figure 4A). With the mosquito syntenin antibody, we observed a moderate reduction in cellular levels and a strong decrease in secreted quantity for syntenin following syntenin RNAi depletion (Figures 4B–E; Supplementary Figures S9A, B). Although the hCD63 antibody detected a band around the expected size (27 kDa for Tsp29Fa and 32 kDa for Tsp29Fb), depletion of neither Tsp29Fa nor Tsp29Fb reduced the amounts of cellular and secreted proteins detected with the hCD63 antibody (Figures 4B–E; Supplementary Figures S9A, B). Of note, we did not detect the hCD63 homolog in naïve media. Our results indicate two potential markers for mosquito EVs.

### 3.5 Separation of EV markers using differential centrifugation and SEC

**FIGURE 5:**
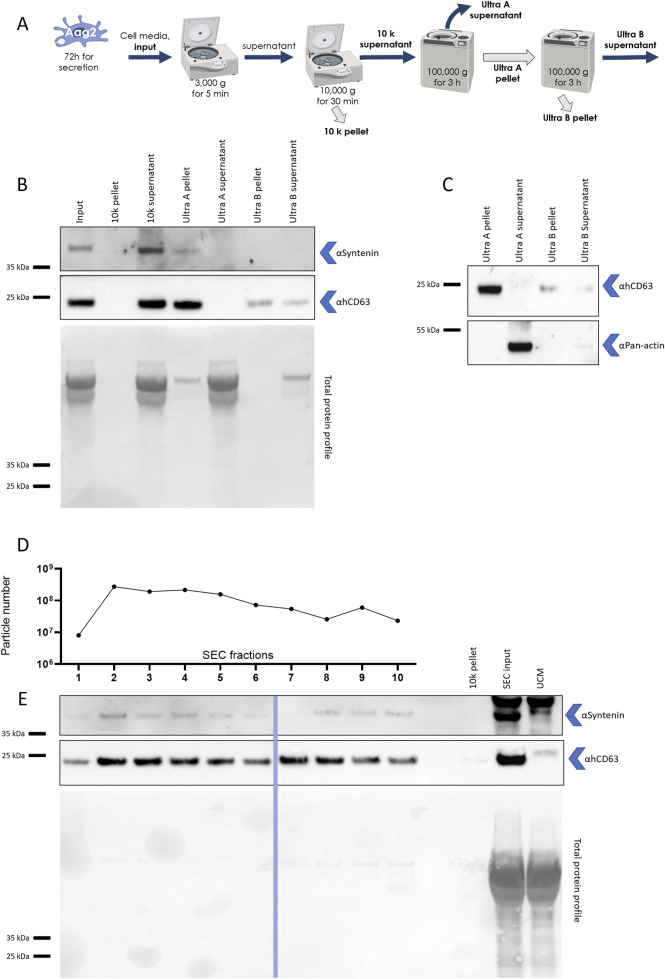
Mosquito syntenin and hCD63 homolog are separated using ultracentrifugation and SEC. **(A)** Scheme of the differential centrifugation design. Pellets and supernatants analyzed by WB are boldened. **(B)** WB detection of syntenin and hCD63 in pellets and supernatants. Red ponceau show the total protein profile. **(C)** WB detection for hCD63 and pan-actin in Ultra A and B pellets and supernatants. **(D)** Particle number in the SEC fractions (1–10) as quantified by NTA. **(E)** WB detection of syntenin and hCD63 in SEC fractions (1–10), 10 K pellet, SEC input (supernatant after 10,000 g centrifugation), and unconditioned medium (UCM). Red ponceau show the total protein profile. The image combines pictures from two different gels, separated by the line.

To further characterize mosquito syntenin and hCD63 homologs as EV markers, we implemented differential centrifugation and SEC technologies, for which we previously described the capacity to separate mosquito EVs according to their size (Figure 3). The analyzed cell media was collected 72 h post initial media replacement. WB detection of the two markers across the different steps of differential centrifugation (Figure 5A) showed that mosquito syntenin and hCD63 homologs were not pelleted at 10,000 g (see 10 k pellet) but remained in the resulting supernatant (see 10 k supernatant) together with most of the total protein content (Figure 5B). In contrast, a first ultracentrifugation at 100,000 g pelleted both markers (see ultra A pellet) and clearly depleted the markers from the resulting supernatant (see Ultra A supernatant), where most of the total protein content remained (Figure 5B). A second ultracentrifugation on the resuspended pellet from the first ultracentrifugation was less efficient at pelleting hCD63 homologs and syntenin (see ultra B pellet); however, most of the total protein remained in the resulting supernatant (see ultra B supernatant) (Figure 3B). To further support the isolation of the putative EV markers by ultracentrifugation compared to non-vesicular proteins, we repeated WB with the anti-pan-actin antibody in pellets and supernatants from the successive ultracentrifugations. Whereas hCD63 homologs were enriched in ultracentrifugation pellets, pan-actin remained in the supernatants (Figure 5C), showing selective concentration in EV markers.

We analyze the distribution of the two markers across the 10 SEC fractions. As mosquito syntenin and hCD63 homologs were not found in pellets resulting from 10,000 g centrifugation, we performed the SEC on cell media depleted of the 10,000-g pellet. High numbers of particles were eluted with each fraction, as determined by NTA (Figure 5D). Both syntenin and hCD63 homologs were detected in each of the fractions (Figure 5E). Interestingly, the EV markers were not associated with total protein levels, which were depleted in the SEC fractions compared to the input (see SEC input) (Figure 5D).

### 3.6 hCD63 homologs are present on the surface of small EVs

**FIGURE 6:**
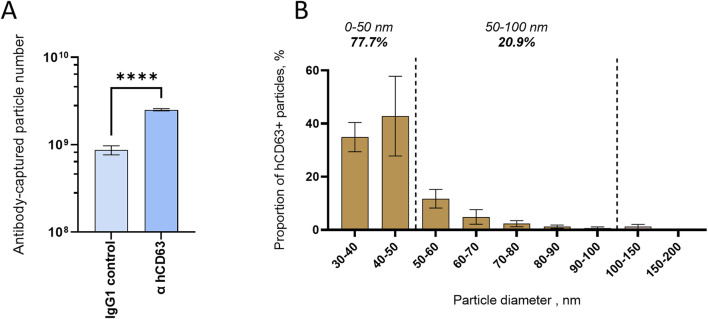
Size distribution of mosquito EVs immuno-captured with hCD63. **(A)** Number of vesicles captured by hCD63, compared to control IgG. **(B)** Size distribution of hCD63-captured vesicles. **(A, B)** Precleared cell media collected at 72 h post media change was used. Bars indicate mean ± s.e.m.

To further characterize hCD63 homologs and the associated EVs, we applied a novel technology combining EV immunocapture and interferometry-based size analysis, encompassed in the Leprechaun system (Unchained lab). First, we found that the hCD63 antibody captured more vesicles than IgG control (Figure 6A), indicating both the ability of the antibody to recognize the native form of the mosquito homolog and the outer position of the targeted epitope. Second, we observed that hCD63-captured vesicles ranged from 30 to 200 nm and were enriched in EVs smaller than 50 nm, which comprised 77.7% of captured EVs (Figure 6B). The Leprechaun technology improved the characterization of the hCD63 homolog marker.

## 4 Discussion

Our study aimed at determining size distribution, evaluating separation methodologies, and identifying markers for mosquito EVs, using *Aedes aegypti* as a model. First, by combining different microscopic technologies, we detected the presence of EVs in mosquito cell media and saliva. Although mosquito EVs ranged in diameter from 20 to 500 nm, the majority was smaller than 100 nm in cell media and smaller than 50 nm in mosquito saliva. Second, we showed that the widely used differential centrifugation methodology separated large (i.e., >100 nm) from smaller EVs (i.e., <100 nm); however, few large EVs remained in ultracentrifugation pellets. In contrast, SEC was unable to segregate EVs by size but could be used to remove protein contaminants for EV purification. Finally, we identified a mosquito homolog of hCD63 as a transmembrane marker and mosquito syntenin as a putative EV luminal marker. Together, these results build the necessary knowledge and tools to promote the study of mosquito EV functions (Reyes-Ruiz et al., 2019; Reyes-Ruiz et al., 2020).

We used multiple microscopic technologies to characterize EVs secreted from mosquito cells and in mosquito saliva. Our cryo-EM observation of lipid-bilayer vesicles, together with previous observations of MVB in mosquito cells (Reyes-Ruiz et al., 2019), indicates that mosquitoes, as most organisms, secrete EVs. Mosquito EVs had an average diameter of 112 nm as determined with NTA, 82 nm with TEM, and 115 nm with cryo-EM. Previous studies using EVs secreted from *Aedes albopictus* cells (i.e., C6/36) showed a smaller diameter with an average of 46 nm as determined with TEM, 55 nm with AFM, and 42 nm with dynamic light scattering (Reyes-Ruiz et al., 2019). Both our results and those of other studies using the *A. albopictus* cell line (Jeppesen et al., 2019) showed that a majority of mosquito EVs were smaller than 100 nm, fitting in the expected size range for mammalian exosomes. Overall, EVs from mosquito cells were similar in size albeit slightly smaller than human EVs, which have an average diameter of approximately 130 nm (Vestad et al., 2017) or 150 nm (Bellotti et al., 2021) as measured with NTA. In mosquito saliva, however, we observed smaller EVs ranging from 20 to 150 nm, with a mode between 30 and 50 nm as measured with TEM. The size of salivary EVs differs with our previous findings where EVs from *A. aegypti* saliva had an overall larger diameter ranging from 100 to 800 nm, as measured with TEM (Yeh et al., 2023). This difference may partially stem from the ultracentrifugation step that we performed in the current study as high centrifuge forces can damage EVs (Mol et al., 2017).

Each of the microscopic technology we used introduced a bias in size quantification. This is reflected in the variation we observed in the average sizes for the same EV preparations analyzed by different technologies (Supplementary Figure S10). NTA leverages the diffusion coefficient to calculate the hydrodynamic radius of a particle. As EVs are negatively charged, their hydrodynamic radius encompasses the particle itself plus the exclusion zone resulting from the solvent repulsed by electrostatic forces (Intermolecular and Surface Forces and Israelachvili, 2011). Likewise, non-vesicular extracellular objects derived from buffer solutions or column matrices can be detected with NTA (Hoover and Murphy, 2020), but these were carefully evaluated as background and subtracted from our analyses. Aside from this bias, NTA is a high-throughput technology that enabled us to analyze millions of particles, providing confidence in the results. NTA has a limit of detection approximately 50 nm for EVs, which precluded the observation of small mosquito EVs. In contrast, TEM and cryo-EM have a much lower limit of visualization but are not high-throughput methods as we sampled EVs to evaluate size distribution. TEM technology dries EVs, collapsing their lumen cargo and producing smaller typical cup-shaped vesicles. Cryo-EM enables detailed observation of the EV limits as defined by lipid bilayers and revealed few EVs with unexpected noncircular shapes that may result from the ultracentrifugation pressure (Akbar et al., 2022). Finally, we used AFM to provide a topographic description of mosquito EVs. Although AFM determines the 3D structure (Sajidah et al., 2022), immobilization of EVs on coated surface may not retain all EVs. To compensate for the different technical biases, we combined complementary technologies, as advised in MISEV guidelines (Théry et al., 2018).

The study of the different EV types requires the development of separation methodologies (Théry et al., 2018). We evaluated two widely used methodologies, namely, differential centrifugation and SEC. Differential centrifugation clearly segregated EVs based on size, whereas SEC was effective in purifying EVs by removing protein contaminants but not amenable to separate EVs by size. Our observations concerning the separation methodologies applied to mosquito EVs are coherent with studies with human EVs (Brennan et al., 2020).

Markers are important to detect EVs. We characterized syntenin as a putative luminal EV marker and a tetraspanin homolog of hCD63 as an extravesicular marker. We reported that *A. aegypti* syntenin conserved both its functional domain sequences and its putative interacting partners as compared to human, suggesting functional homology. We then validated the target of an anti-mosquito syntenin antibody. For hCD63 homologs, we selected two phylogenetically related mosquito tetraspanins (i.e., Tsp29Fa and Tsp29Fb) and used an hCD63-targeted antibody to detect a protein of the expected size for the two mosquito homologs. Previous studies proposed Tsp29Fb as the homolog of hCD63 using a different antibody raised against a different peptide sequence (Vora et al., 2018). However, with our hCD63 antibody, we could not differentiate between the two homolog candidates using loss-of-function studies. Nonetheless, both syntenin and hCD63 markers were associated with EVs when using differential centrifugation and SEC separation. Finally, using the hCD63 antibody for immunocapture with the Leprechaun system, we showed that a part of the mosquito homolog localizes on the outer part of EVs, as expected for tetraspanins, and described the size distribution of the associated EVs. Although our initial characterization of two mosquito EV markers is informative, a third marker is recommended in MISEV guidelines (Théry et al., 2018). This could be addressed by previous identifications of antibodies against mosquito tetraspanin homologs of hCD9 and hCD81 (Reyes-Ruiz et al., 2019); however, the antibody targets will need to be validated.

## 5 Limitations of the study

Although we used several microscopic technologies and separation methodologies, other approaches remain to be tested to provide a stronger baseline for mosquito EV studies. For instance, density gradient separation (Brennan et al., 2020) and high-resolution flow cytometry (van der Vlist et al., 2012) have never been applied to mosquito EVs. These tools provide complementary information about EV density and size distribution, and can be used to isolate distinct EV populations. Although we developed our EV isolation protocol based on mosquito and tick studies (Zhou et al., 2018; Vora et al., 2018; Reyes-Ruiz et al., 2019), the second ultracentrifugation seemed too harsh as the hCD64 marker was recovered in the supernatant. Increased speed and time significantly influence soluble protein isolation, EV yield, and diameter (Cvjetkovic et al., 2014). The development of tools will be necessary to reveal commonalities and differences between arthropods and mammalian EVs, and, specifically, to study the impact of viral infections on mosquito EVs. Additionally, the *A. aegypti* cell line we used as a model for mosquitoes may not represent the diversity of EVs from different mosquito species. Although we compared our results with prior reports using EVs secreted by another *Aedes* cell line, other cell lines from different mosquito species, such as U4.4 from *Ae. albopictus* and Hsu from *Culex quinquefasciatus*, would complete the knowledge and tool library for the study of mosquito EVs. Finally, we were surprised by the size difference observed between EVs secreted from cell line and in saliva. Our results highlight the limits of *in vitro* models, which nonetheless remain indispensable to study EVs as minute quantities of saliva preclude the application of most biochemical approaches.

## Data Availability

The original contributions presented in the study are included in the article/Supplementary material, further inquiries can be directed to the corresponding author.
