# Supplementary material for: Characterization of size distribution and markers for mosquito extracellular vesicles
Source: Front Cell Dev Biol. 2025 Apr 11;13:1497795. doi: 10.3389/fcell.2025.1497795 (PMC12021844; doi:10.3389/fcell.2025.1497795)
Supplement: Supplementary file 1 [file DataSheet1.docx]

**Supplementary information**


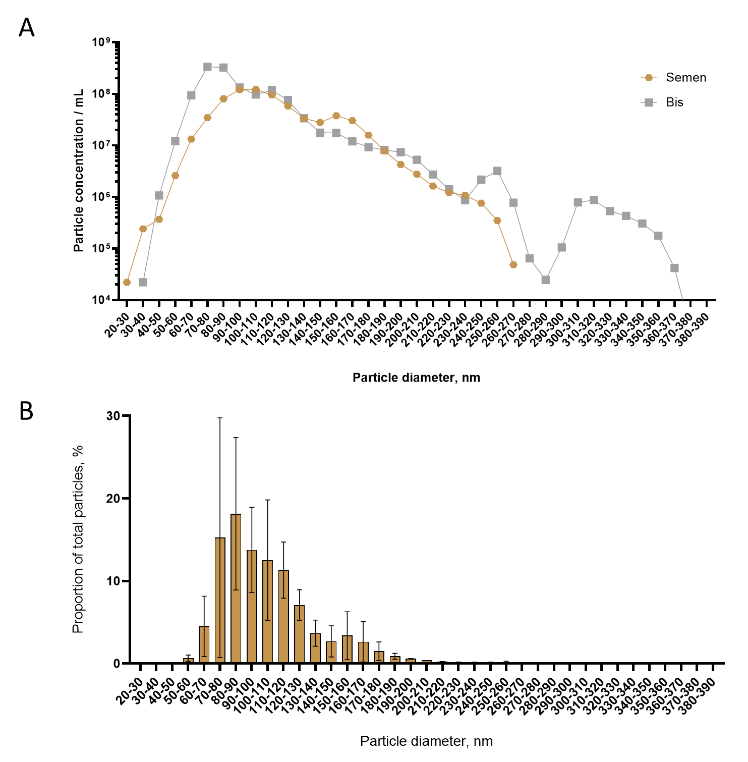


**Figure S1. Detection of particles by NTA in unconditioned media (UCM).** **A.** Quantification of total particles. Total particle concentration: Semen, 6.93 x 10^8^ / mL; Bis, 1.32 x 10^9^ / mL. **B.** Size distribution.


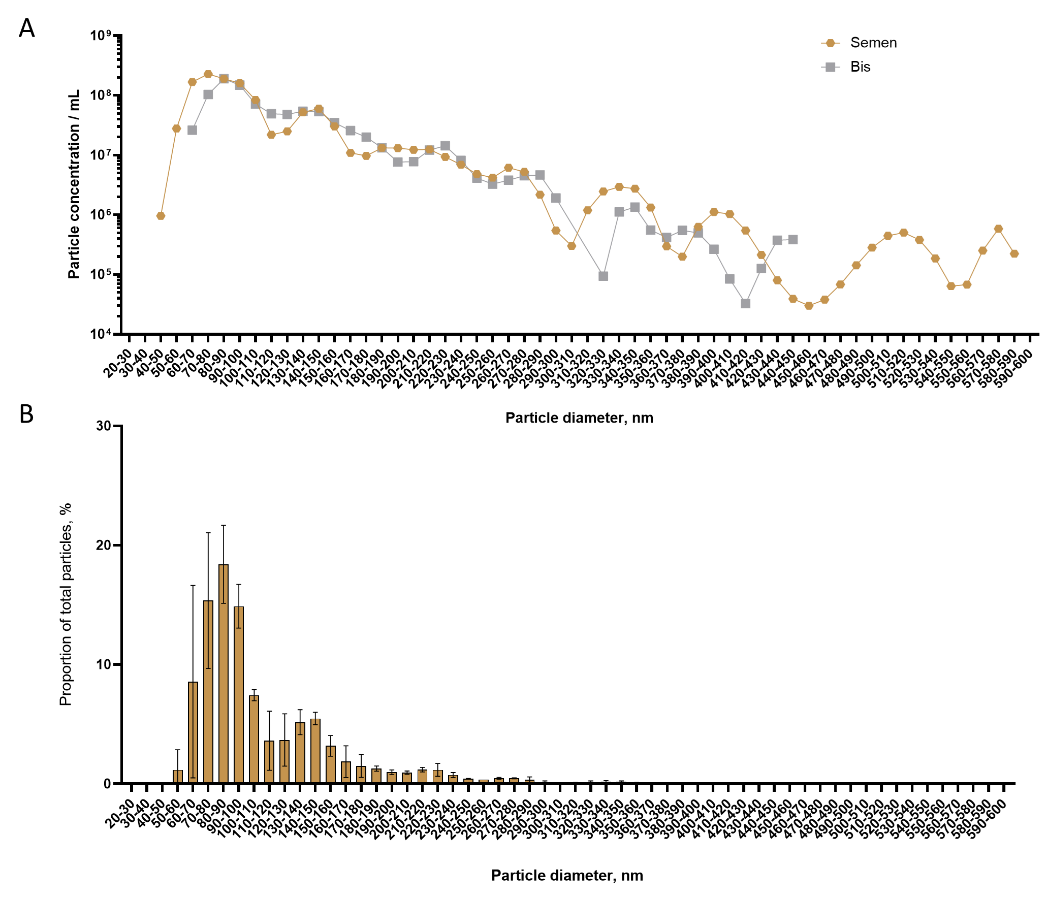


**Figure S2. Details of size distribution with NTA for particles released from mosquito cells.** **A.** Size particle distribution for the two repeats. Total particle concentration: Semen, 1.92 x 10^9^ / mL; Bis, 2.18 x 10^9^ / mL. **B.** Size distribution.


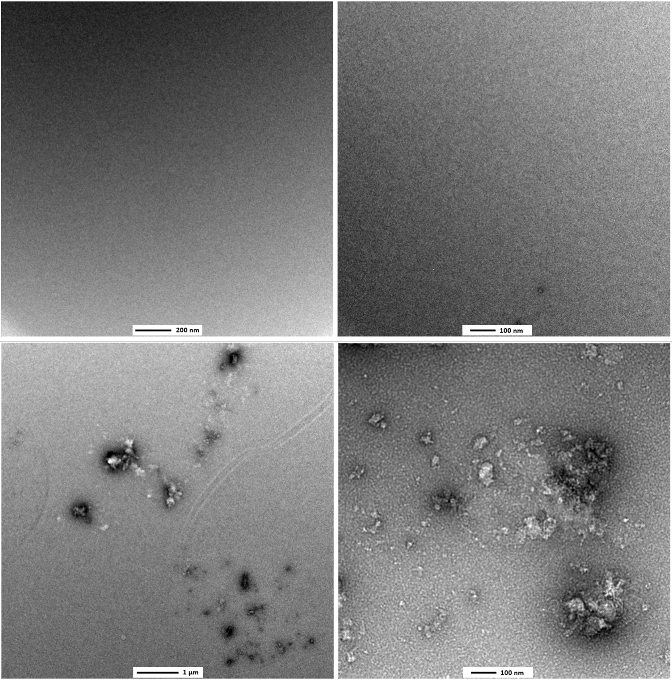


**Figure S3. Unconditioned media analysis by TEM.** Representative pictures are shown.


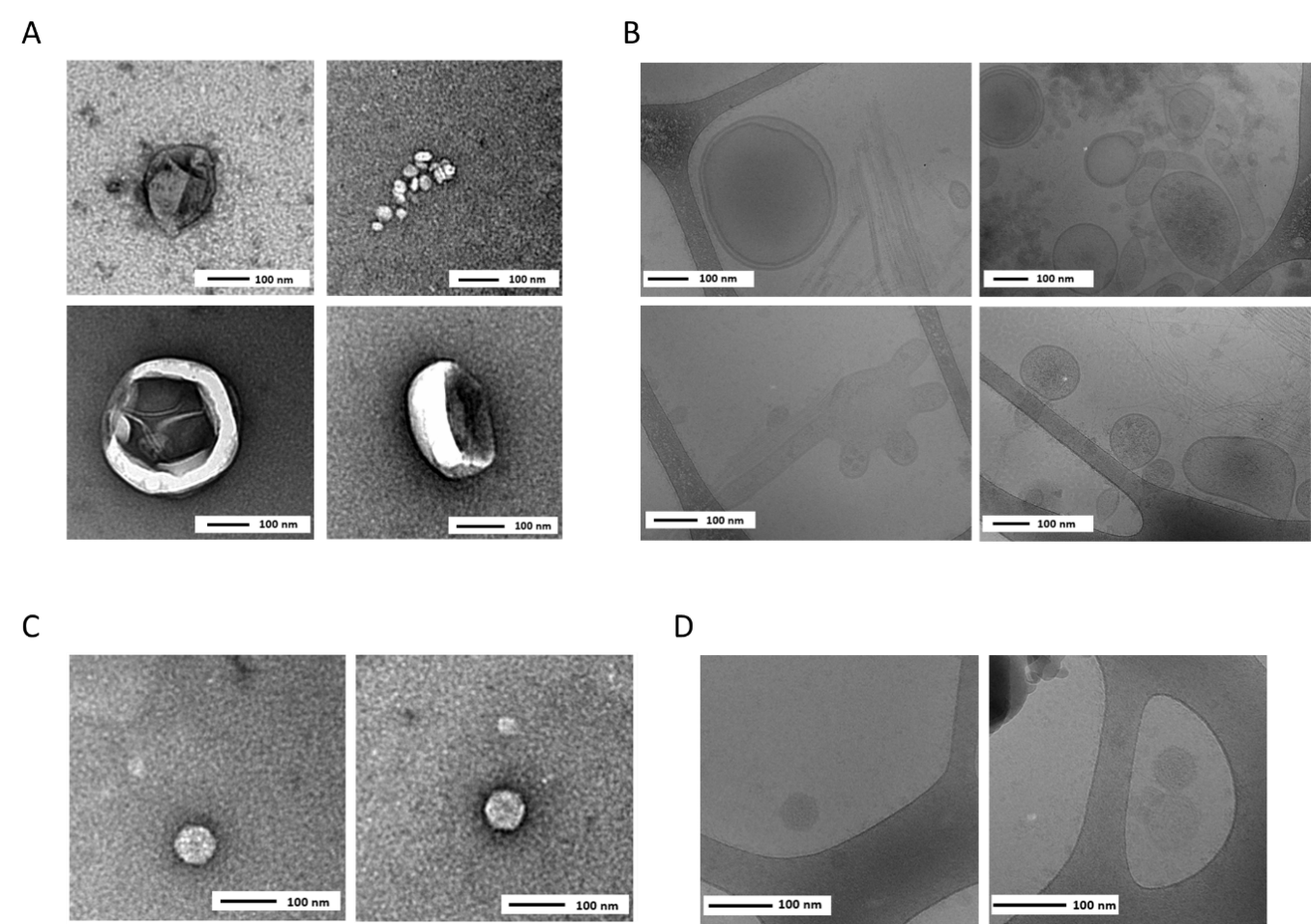


**Figure S4. TEM and Cryo-EM observations of particles in ultracentrifugated cell media.** **A,B.** Representative pictures of EVs observed by TEM (A) and Cryo-EM (B). **C, D.** Putative viral particles observed by TEM (C) and Cryo-EM (D).


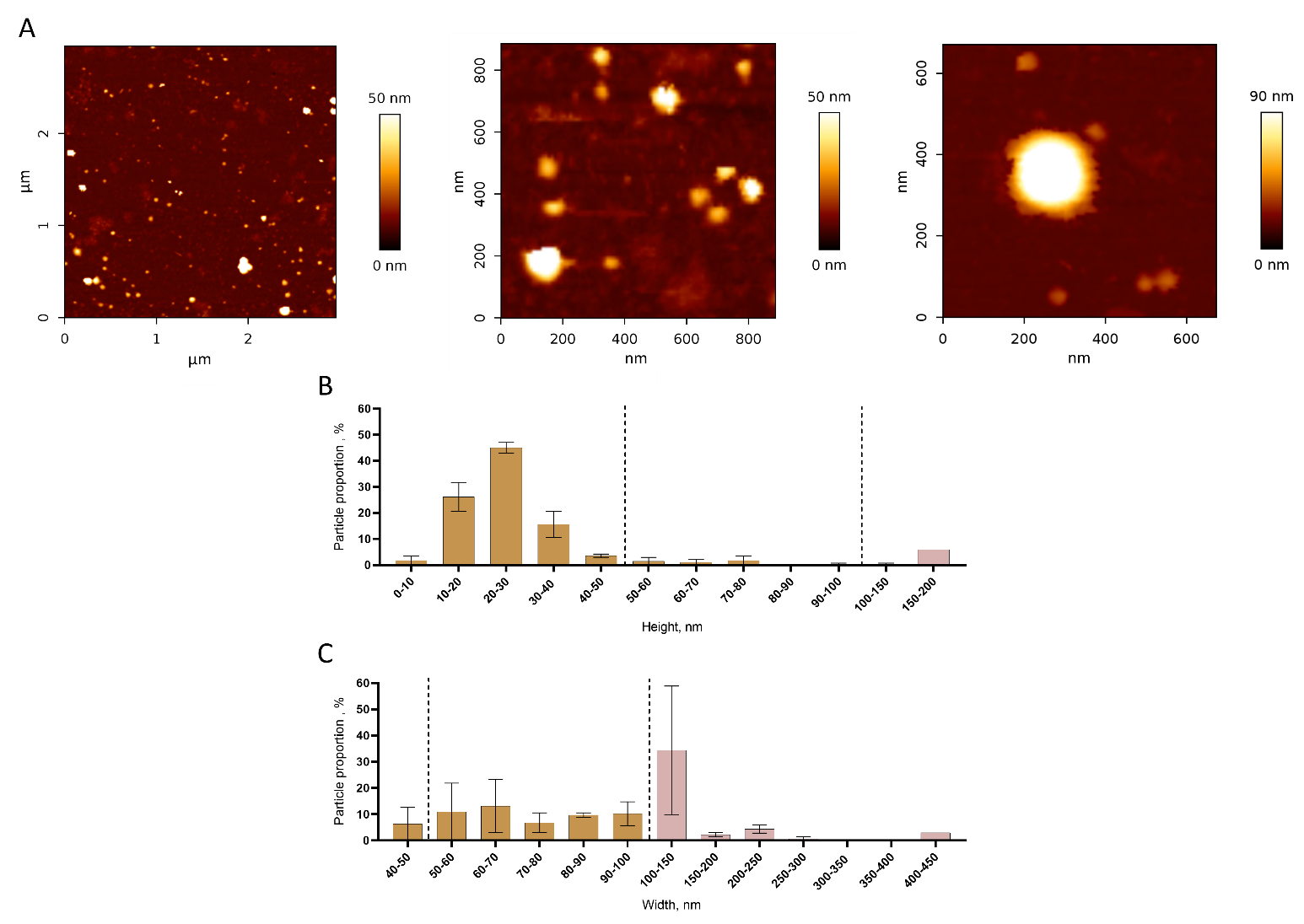


**Figure S5. Size distribution of particles released from mosquito cells as determined by AFM. A.** Representative pictures. **B, C.** Distributions of particles height (B) and width (C). Bars indicate mean ± s.e.m. N analysed EVs, 176 from 2 biological repeats.


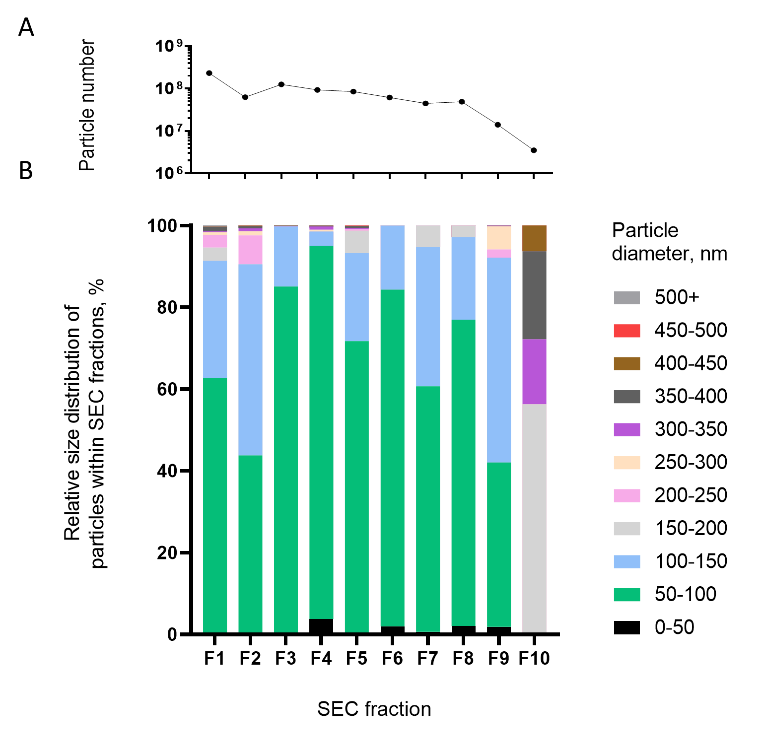


**Figure S6. Repeat of the SEC analysis.** **A, B.** Particle number per fractions of SEC (A) and relative size distribution of particles in each fraction (F1-F10) (B) measured by NTA.


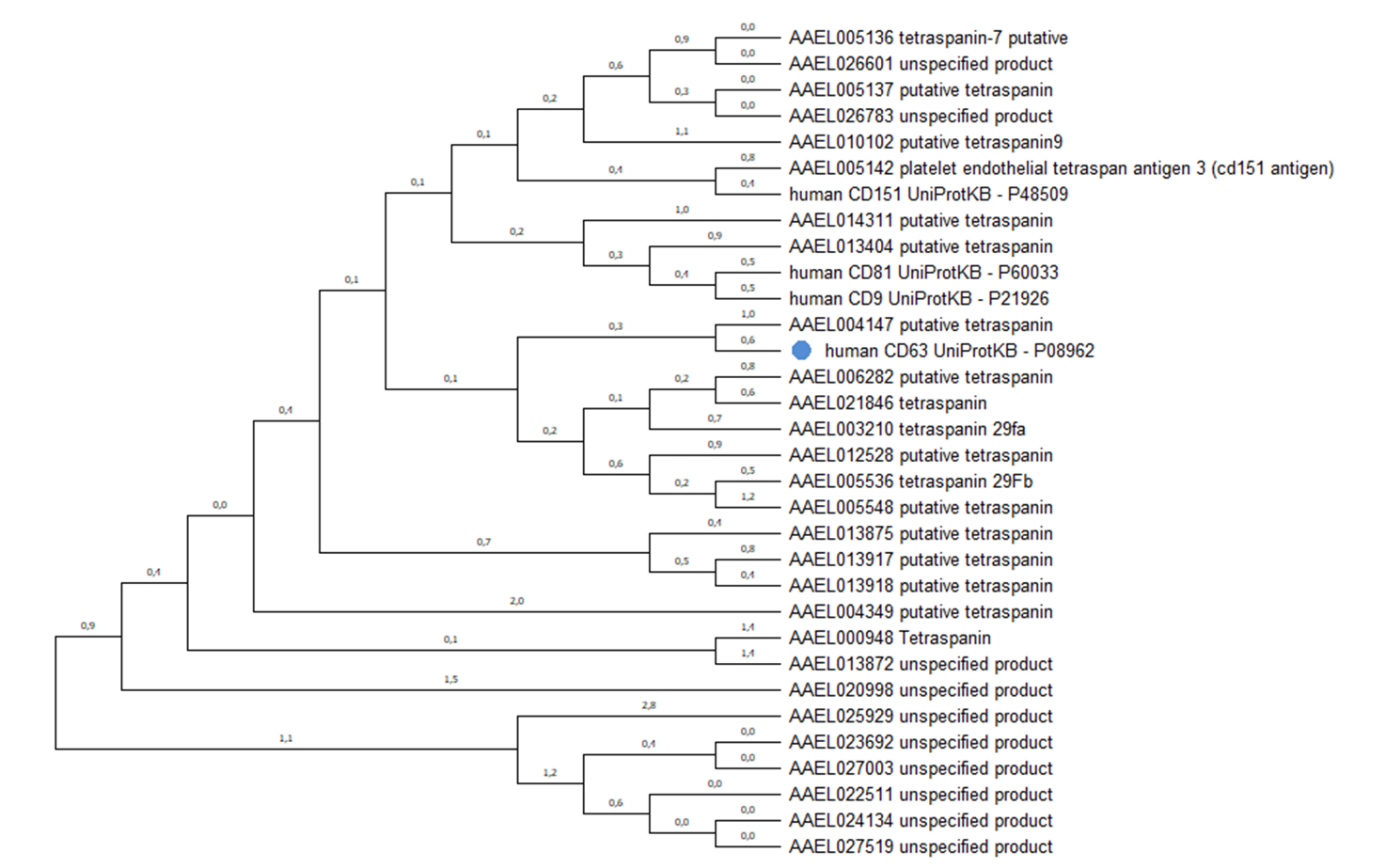


**Figure S7. Maximum likelihood amino acid tree for *Aedes aegypti* and human tetraspanins.** Human CD63 protein is indicated by a blue circle. Bootstraps are shown above branches.


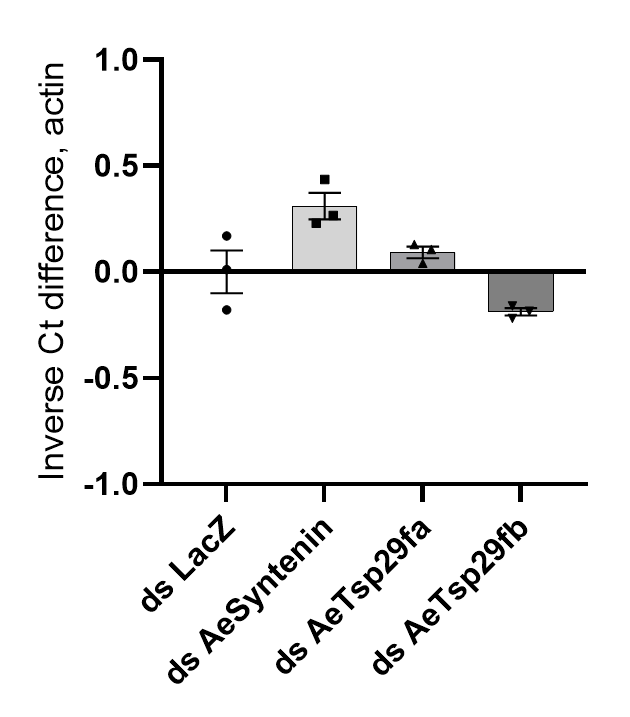


**Figure S8. Evaluation of cell survival upon depletion of syntenin, Tsp29fa and Tsp29fb.** Inverse Ct difference for Actin expression is shown. Bars indicate average ± sem.


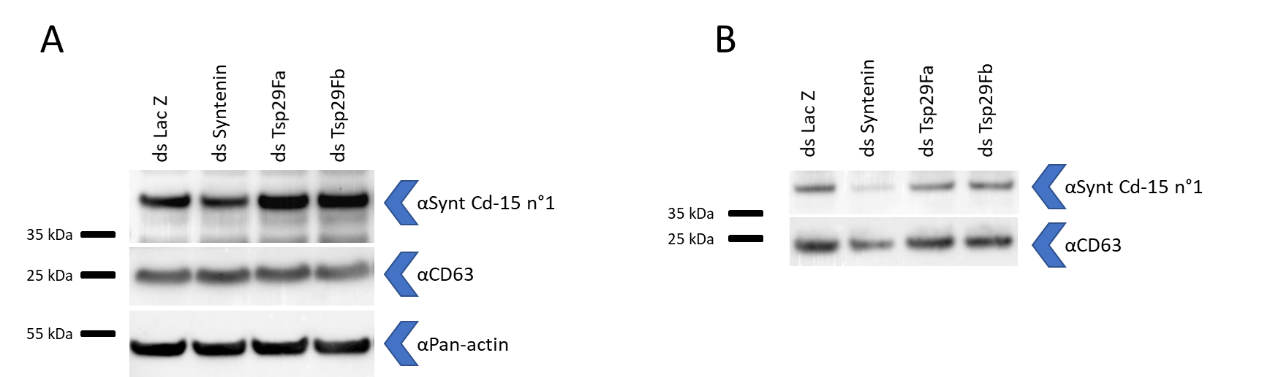


**Figure S9. Second repeat of syntenin and hCD63 detection after dsRNA transfection. A, B.** WB of syntenin and hCD63 in cell lysate (A) and cell media (B) at 72h post transfection with dsRNA.


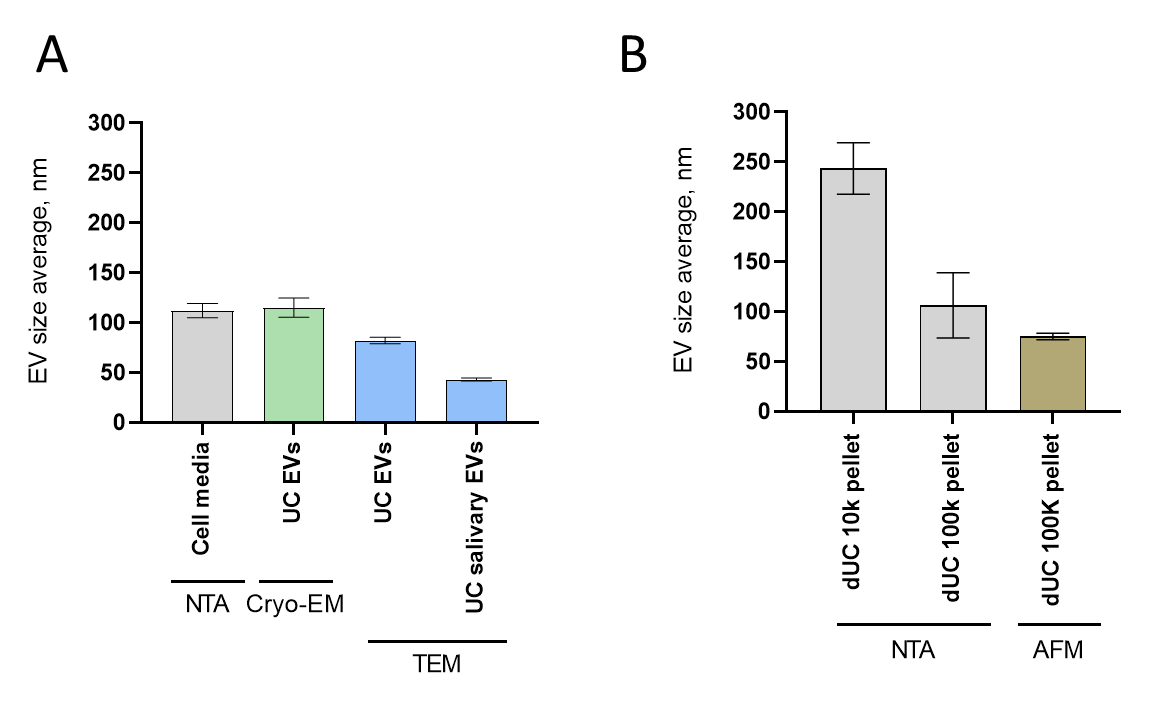


**Figure S10. EV sizes according to separation and analytical methods. A, B.** Averages of EV size from cell media, ultracentrifugated (UC) cell media and UC salivary EVs (A) and from differential centrifugation of cell media (B) as determined by either NTA, Cryo-EM, TEM or AFM. Bars indicate average ± sem.

**Table S1**: Primer list.

| **Gene** | **ID** | **dsRNA primers** | **qPCR primers** |
| --- | --- | --- | --- |
| LacZ (dsRNA control) | | taatacgactcactatagggACACCAACGTGACCTATCCC |  |
|  |  | taatacgactcactatagggCCGCCACATATCCTGATCTT |  |
| Tsp29fa | AAEL003210 | taatacgactcactatagggCGGCAAACAAGCGTTTTAGA | GCATGACGTTGACGTTTCGAT |
|  |  | taatacgactcactatagggAGCATCCGAAGAATGCAATG | TGGAGCTTTGCAAGGTGTTG |
| Tsp29fb | AAEL005536 | taatacgactcactatagggGACCGGAACGACCTCAACA | CGCCGGCCCACCTGAT |
|  |  | taatacgactcactatagggCATGGCGGTGCTTTCCTTGA | GAGTTCGGCGAACCATCTCCT |
| Syntenin | AAEL005391 | taatacgactcactatagggGAGACAGCCAAAAAGCAGGA | CACGATCCATAAAAACGCGTGA |
|  |  | taatacgactcactatagggCAACTCTCGAATTCCGTTGG | ATGCTTTTGCGGCAATTGGT |

**Table S2. Identification of the main syntenin partners in *Aedes aegypti* genome**

| Human protein name | Uniprot ID (human) | Vector base ID (*Aedes aegypti*) | Identity  (%) | Similarity  (%) | Coverage  (%) | E value |
| --- | --- | --- | --- | --- | --- | --- |
| Syndecan 1 | P18827 | AAEL013969 (Syndecan) | 50 | 70 | 45 | 3e-19 |
| Syndecan 2 | P34741 | AAEL013969 (Syndecan) | 50 | 64 | 58 | 9e-17 |
| Syndecan 3 | O75056 | AAEL013969 (Syndecan) | 49 | 64 | 14 | 7e-19 |
| Syndecan 4 | P31431 | AAEL013969 (Syndecan) | 35 | 56 | 44 | 6e-18 |
| CD63 | P08962 | AAEL005536 (Tetraspanin 29fb) | 28 | 51 | 79 | 1e-31 |
| CD63 | P08962 | AAEL003210 (Tetraspanin 29fa) | 33 | 51 | 96 | 8e-47 |
| c-Src | P12931 | AAEL004592 (Tyrosine kinase) | 50 | 67 | 91 | 0 |
| ARF6 | P62330 | AAEL002311 (ADP-ribosylation factor) | 37 | 58 | 84 | 2e-39 |
| Tsg101 | Q99816 | AAEL012515 (Tumor suppressor protein) | 49 | 65 | 78 | 3e-126 |
| ALIX | Q8WUM4 | AAEL011271 (programmed cell death 6-interacting protein) | 40 | 59 | 97 | 0 |
